# Effect of clove powder on quality characteristics and shelf life of kimchi paste

**DOI:** 10.1002/fsn3.833

**Published:** 2019-01-24

**Authors:** Miran Kang, JeongSun Park, SeungRan Yoo

**Affiliations:** ^1^ Hygienic Safety and Analysis Center World Institute of Kimchi Gwangju Korea; ^2^ Industrial Technology Research Group World Institute of Kimchi Gwangju Korea

**Keywords:** clove, fermented food, kimchi paste, quality, shelf life

## Abstract

Clove has been shown to extend the shelf life of various foods. This study investigated whether it can prolong the shelf life of kimchi paste. Clove powder at concentrations of 0%, 0.5%, 1%, 1.5%, and 2% was added to kimchi paste, which was then sealed and stored at 10°C for 20 days. Changes in microbial counts, gas composition, sugar and organic acid contents, pH, titratable acidity, and reducing sugar content were evaluated. Adding clove powder inhibited the growth of total aerobic and lactic acid bacteria and delayed changes in O_2_ and CO_2_ concentration and sugar and organic acid contents. It also slowed the decrease in pH, increase in titratable acidity, and changes in reducing sugar content. These results indicate that clove powder effectively prolongs the quality attributes and thus extends the shelf life of kimchi paste.

## 1 INTRODUCTION

Kimchi is a widely consumed traditional Korean food that is produced by blending pastes and several vegetables such as cabbage, radish/young radish, green onion, mustard leaf, cucumber, garlic chive, and perilla leaf, followed by fermentation by natural lactic acid bacteria during storage. Kimchi pastes have many flavors—including pungent, sweet, sour and/or spicy—that are conferred by specific ingredients, including red pepper powder, salt, sugar, fermented and salted seafood, and starch paste (Kim & Kim, 2003; Park et al., 2012).

Recently, demands for distinct kimchi pastes and salted cabbages have sharply increased in Korea due to consumer interest in making kimchi at home according to personal preferences. As such, commercial production of kimchi paste has increased. However, its short shelf life is an obstacle for mass production since kimchi is manufactured through a nonthermal process that can lead to the growth of pre‐existing microorganisms and thereby present a risk to consumer health. In particular, microbiological and enzymatic activities after production decrease pH, leading to formation of excessive organic acids and CO_2_ and consequent deterioration of kimchi paste and packaging (Cheon, Seo, Chung, & Chun, 2016; Kang, Jung, & Seo, 2015; Park et al., 2008).

The ideal way to overcome the problem of over‐ripening is to control lactic acid bacterial growth without damaging the product (Ko, Kim, & Park, 2012; Song et al., 2004). Several studies have investigated ways of extending the shelf life of kimchi or kimchi paste using an antimicrobial agent (Ko et al., 2012), chitosan (Seo, Bang, & Jeong, 2004), ethanol or organic acid (Kim & Hahn, 2003), gamma radiation (Song et al., 2004), glucono‐δ‐lacton (Han & Kang, 2004), heat treatment (Hong, Cheigh, & Lee, 2006), medicinal herb extracts (Lee, Cho, Choi, & Kim, 1998), natural preservatives (Moon, Byun, Kim, & Han, 1995), heat treatment and nisin·yucca extract (Kim et al., 2010), and oyster shell powder (Choi, Whang, Kim, & Suh, 2006). However, these approaches have met with limited success.

Cloves are used as a topical analgesic that has been used to promote healing and prevent aging, and treat cardiovascular disease, arthritis, infections, digestive problems, skin cancer, and thyroid dysfunction; it is also used in fragrances and flavorings (Chaieb et al., 2007; Fu et al., 2007; Nassar et al., 2007). Clove extracts in the form of essential oil and powder also exhibit a wide range of biological effects, including antioxidant and antibacterial activities (Menon & Garg, 2001; Sultana, Anwar, Mushtaq, Aslam, & Ijaz1, 2014). Cloves may extend the shelf life of various foods (meat, baked goods, etc.) by suppressing the growth of foodborne pathogens while adding a characteristic flavor (Ibrahium, Abd El‐Ghany, & Ammar, 2013; Khaleque et al., 2016; Kumar & Tanwar, 2011; Tajik, Farhangfar, Moradi, & Rohani, 2014). However, no studies to date have investigated the effects of clove powder on the microbiological and physiochemical qualities of kimchi paste.

The present study addressed this issue by adding clove powder to kimchi paste and evaluating microbial count, headspace composition including CO_2_, free sugar and organic acid contents, pH, titratable acidity, and reducing sugar content during fermentation.

## 2 MATERIALS AND METHODS

### 2.1 Sample preparation

Dried cloves were purchased from online markets and ground into a fine powder using an HR1372 grinder (Philips, Huizhou, China). Commercial kimchi paste was purchased from a local market in Gwangju, Korea; the composition (w/w) is shown in Table 1. The paste was divided into five groups. The first was left untreated to examine baseline quality characteristics, and clove powder (0.5%, 1%, 1.5%, and 2%) was added to the remaining treatment groups. Each group of kimchi paste was placed on a plastic tray (130 × 95 × 60 mm) and sealed with plastic film using an MS2 sealing machine (Packsis, Yangju, Korea), and stored at 10°C for 20 days.

**Table 1:** Composition (w/w) of kimchi paste

Ingredient	Content (%)
Radish	31.6
Red pepper powder	15.8
Seaweed stock	7.4
Salt‐fermented anchovy	10.8
Salted shrimp sauce	6.3
Ground garlic	5.4
Glutinous rice paste	4.7
Onion	3.2
Green onion	3.2
Dried red pepper	2.8
Pear juice	2.2
Chives	1.6
Ground ginger	1.6
Sugar	1.6
Leaf mustard	0.9
Salt‐fermented croaker	0.9

### 2.2 Determination of moisture content and salinity

The moisture content of samples was determined using an MB45 moisture analyzer (Ohaus, Greifensee, Switzerland). About 3 g of sample were placed in the sample holder and dried until a constant weight was obtained. Salinity was measured by Mohr's titration (Chen, Hsieh, Weng, & Chiou, 2005). Samples (1 g) were homogenized in 100 ml distilled water and then filtered through Whatman No. 2 filter paper (Whatman, Springfield, UK). A 1 ml volume of 2% potassium chromate indicator was added to 10 ml of the filtered sample solution, and the mixture was titrated against 0.02 N AgNO_3_ until the end point (a red‐brown color) was reached.

### 2.3 Scavenging of 2,2‐diphenyl‐1‐picrylhydrazyl (DPPH) radical

The assay was carried out in a flat‐bottomed 96‐well plate as previously described (Chatatikun & Chiabchalard, 2013), with slight modifications. Clove powder (100 mg) and freeze‐dried kimchi paste samples (100 mg) were extracted with 100 ml ethanol. Different concentrations of clove (0.125, 0.25, 0.5, and 1.0 mg/ml), kimchi paste sample (1 mg/ml), or standard ascorbic acid (100 μg/ml) were added to 100 μl of 0.2 mM methanolic DPPH solution in the 96‐well plate. After mixing, the plate was incubated for 30 min at room temperature in the dark. The absorbance of each well was measured at 517 nm with a SPECTRO Star Nano spectrophotometer (BMG Labtech, Ortenburg, Germany). Percent inhibition was calculated with the following equation.

$$\%\text{Inhibition}=\frac{A_{\mathrm{control}}-A_{\mathrm{sample}}}{A_{\mathrm{control}}}\times 100$$

### 2.4 Microbiological analysis

Samples (10 g) were mixed with 90 ml of sterile saline (0.85% NaCl, w/v) in a sterile stomacher bag and then homogenized using a MIX 2 blender (AES Laboratoire, Combourg, France) for 1 min, filtered through sterile cheesecloth, and diluted with sterile saline to determine microbial count. Serial dilutions were prepared in triplicate. For total aerobic bacteria counts, samples were plated onto 3M Petrifilm aerobic count plates (3M Co., Saint Paul, MN, USA) and incubated at 37°C for 48 hr. For lactic acid bacteria counts, diluted samples were plated onto modified de Man, Rogosa& Sharpe agar (BD Difco, Franklin Lakes, NJ, USA) with 0.2% Bromocresol Purple indicator and 0.01% cycloheximide and incubated at 37°C for 24 hr. Yeast and molds were plated on 3M Petrifilm Yeast and Molds Count Plates (3M Co.) and incubated at 25°C for 72 hr. Coliforms were counted using 3M Petrifilm *Escherichia coli*/Coliform count plates (3M Co.) following incubation at 37°C for 24 hr.

### 2.5 Analysis of packaging headspace gas composition

CO_2_ and O_2_ concentrations in the packaging were measured by applying a septum to the outer surface of three packages per treatment and then inserting a needle sensor attached to a GS3 Micro Headspace Gas Analyzer (Illinois Instruments, Ingleside, IL, USA) through the package seal.

### 2.6 Extraction and analysis of sugars and organic acids

Kimchi paste (5 g) was homogenized in 20 ml of distilled water and then passed through an 8‐μm cellulose filter (Whatman) followed by a 0.45‐μm polyvinylidene difluoride syringe filter (Agilent, Santa Clara, CA, USA). The filtrate was diluted and injected into a Dionex 3000 high‐performance liquid chromatography system (Dionex, New York, NY, USA). To analyze sugar content, water was used as the solvent at a flow rate of 0.5 ml/min; a Shodex RI‐101 (Kawasaki, Japan) detector was used. Sucrose, glucose, fructose, and mannitol contents were calculated using external standards. The extract used in the sugar analysis was also used for organic acid analysis. Acids were separated using an Aminex 87H column (300 × 10 mm; Bio‐Rad, Hercules, CA, USA), and 0.01 N H_2_SO_4_ was used as an eluent at a flow rate 0.5 ml/min. Acids were detected with an ultraviolet detector operating at 210 nm. External standards were used to calculate lactic, acetic, citric, and malic acid contents.

### 2.7 Determination of pH and titratable acidity

Sample pH was measured using pH meter (Accumet AB 15, Thermo Fisher Scientific, Pittsburgh, PA, USA). The titratable acidity of the kimchi paste was determined according to a method described by the Association of Official Analytical Chemists (2005). Briefly, after homogenizing 10 g of kimchi paste in 90 ml distilled water, the sample was titrated with 0.1 N NaOH solution until the end point (pH 8.3) was reached. Total acidity was calculated as a percentage of lactic acid.

### 2.8 Determination of reducing sugar content

Reducing sugar content was determined according to the 3,5‐dinitrosalicylic acid (DNS) method (Miller, 1959). Each sample (2 ml) was diluted with distilled water and mixed with 2 ml DNS. After boiling at 100°C for 5 min and cooling in ice water, the absorbance at 550 nm was measured using a SPECTRO Star Nano spectrophotometer. The reducing sugar content (mg/g) is expressed as glucose equivalents.

### 2.9 Statistical analysis

Data are expressed as mean ± *SD* of triplicate samples. Each experiment was performed at least three times. Data were analyzed using SPSS v.19.0 software (SPSS Inc., Chicago, IL, USA) by one‐way analysis of variance. Duncan's multiple range test was used to compare the effects of clove powder addition or fermentation period. Differences were considered statistically significant at *p *<* *0.05.

## 3 RESULTS AND DISCUSSION

### 3.1 Initial moisture content and salinity

**Figure 1:**
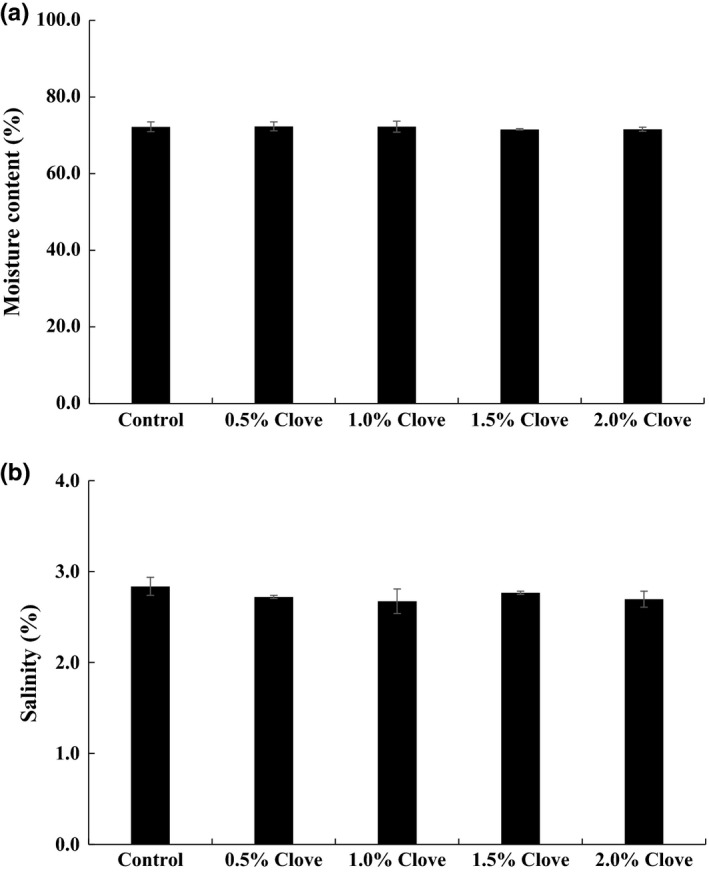
Initial moisture content (a) and salinity (b) of kimchi paste treated with various concentrations of clove powder

There were no differences in moisture content of kimchi paste at the early stage of storage between untreated control sample (72.23%) and samples treated with clove powder (71.56%–72.33%) (Figure 1a). The initial salinity of kimchi pastes was 2.84% for the control and 2.67%–2.77% for treatment groups (Figure 1b).

### 3.2 DPPH radical scavenging activity

**Figure 2:**
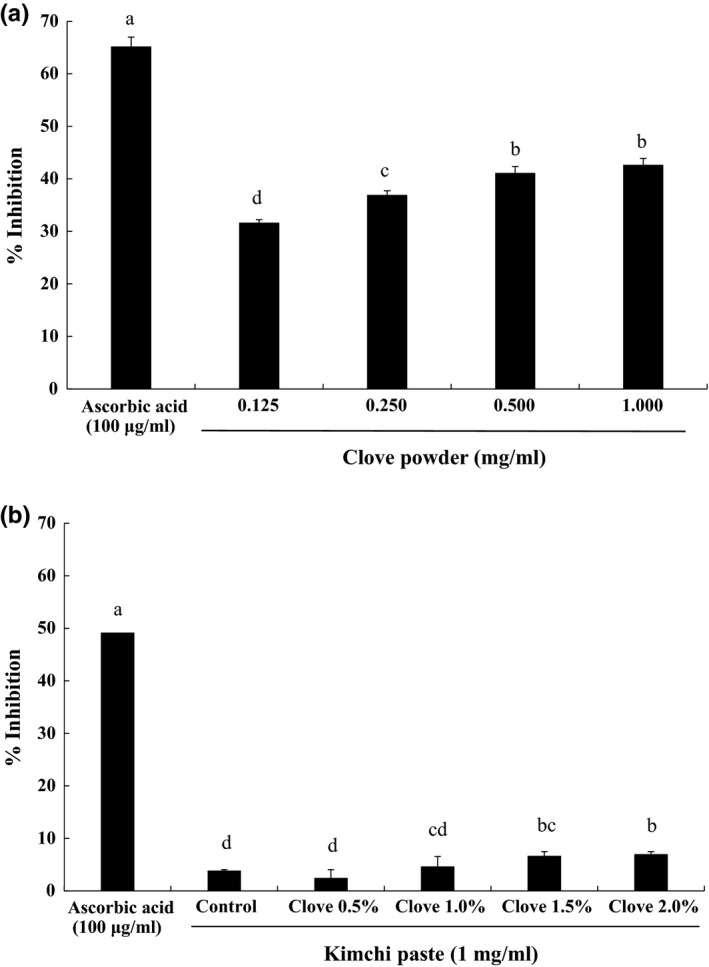
DPPH radical scavenging activities of clove powder (a) and kimchi paste with various concentrations of clove powder (b). Means with different letters differ significantly (*p *<* *0.05)

The major constituent of clove oil and extract is eugenol, a phenolic compound with antioxidant activity (Chaieb et al., 2007; Nassar et al., 2007) that is exerted via mechanisms such as radical scavenging and metal ion chelation. Eugenol also participates in photochemical reactions (Mihara & Shibamoto, 1982). Here, we investigated the DPPH radical scavenging activity of various concentrations of clove powder (0.125–1 μg/ml) added to kimchi paste relative to ascorbic acid (100 μg/ml). Clove powder showed DPPH‐scavenging activity dose‐dependently. Different letters indicate statistically significant (*p *<* *0.05) differences between groups (Figure 2a). The antioxidant activity of the paste increased as a function of clove powder concentration, as evidenced by increased DPPH radical scavenging. Different letters indicate significant (*p *<* *0.05) differences between groups (Figure 2b); extracts showed DPPH scavenging activity at a clove concentration of 1 mg/ml. Many studies have reported linear relationships between total phenolic content and antioxidant capacity in foods (Kalt, Forney, Martin, & Prior, 1999; Sim & Han, 2008).

### 3.3 Microbiological changes during storage

Changes were observed in the microbial composition of kimchi pastes containing various concentrations of clove powder during 20 days of storage at 10°C. The different uppercase letters in the row mean statistically significant (*p *<* *0.05) differences according to fermentation days. The different lowercase letters in the column mean statistically significant (*p *<* *0.05) differences according to concentration of clove powder (Table 2). The initial amount of total aerobic bacteria in the pastes was 6.68 log CFU/g for the control and 6.66–6.75 log CFU/g for treatment groups. After 5 and 15 days of storage, total aerobic bacteria in the paste reached 7.50 and 7.57 log CFU/g, respectively, in the control and 6.67–6.81 and 6.66–6.89 log CFU/g, respectively, in samples with 0.5%–2% clove powder. This is consistent with previous studies reporting that clove oil reduced total aerobic bacteria content in cake (Ibrahium et al., 2013) and buffalo meat (Naveena, Muthukumar, Sen, Babji, & Murthy, 2006). The antibacterial activity is mainly attributable to eugenol; the partially hydrophobic nature of phenolic compounds can induce cell wall degradation, disrupt the cytoplasmic membrane, cause damage to membrane proteins, and interfere with membrane‐integrated enzymes, eventually leading to cell death (Shan, Cai, Brooks, & Corke, 2007).

**Table 2:** Changes in microbial composition (log CFU/g) of kimchi paste containing various concentrations of clove powder during fermentation at 10°C

Sample		Day 0	Day 5	Day 10	Day 15	Day 20
Total bacteria	Control	6.68 ± 0.00^Ec^	7.50 ± 0.02^Ca^	6.81 ± 0.02^Da^	7.57 ± 0.01^Ba^	7.97 ± 0.02^Aa^
	0.5%	6.73 ± 0.02^Dab^	6.80 ± 0.01^Cb^	6.77 ± 0.01^CDb^	6.89 ± 0.06^Bb^	7.98 ± 0.00^Aa^
	1.0%	6.75 ± 0.01^Ca^	6.81 ± 0.00^Bb^	6.73 ± 0.02^CDc^	6.71 ± 0.00^Dc^	7.88 ± 0.04^Aa^
	1.5%	6.66 ± 0.02^Cc^	6.67 ± 0.00^Cd^	6.72 ± 0.02^Bc^	6.66 ± 0.04^Cc^	7.51 ± 0.02^Ab^
	2.0%	6.72 ± 0.01^Ab^	6.76 ± 0.00^Ac^	6.73 ± 0.01^Ac^	6.68 ± 0.02^Ac^	6.60 ± 0.30^Ac^
Lactic acid bacteria	Control	5.60 ± 0.01^Ea^	7.57 ± 0.02^Aa^	6.79 ± 0.01^Ba^	6.76 ± 0.01^Ca^	6.73 ± 0.01^Da^
	0.5%	5.14 ± 0.06^Cb^	5.71 ± 0.03^Ab^	5.50 ± 0.01^Bb^	3.72 ± 0.12^Db^	3.33 ± 0.03^Eb^
	1.0%	4.40 ± 0.02^Cc^	4.47 ± 0.04^Bc^	5.12 ± 0.01^Ab^	3.06 ± 0.06^Dc^	2.90 ± 0.05^Ec^
	1.5%	4.19 ± 0.04^Ad^	3.85 ± 0.01^Bd^	3.62 ± 0.00^Cd^	2.90 ± 0.05^Dcd^	1.39 ± 0.09^Ed^
	2.0%	3.42 ± 0.04^Ae^	3.31 ± 0.08^Ae^	3.46 ± 0.01^Ad^	2.75 ± 0.15^Bd^	1.30 ± 0.09^Cd^
Yeast and molds	Control	5.45 ± 0.04^Aa^	5.57 ± 0.02^Aa^	5.44 ± 0.02^Aa^	5.52 ± 0.03^Aa^	5.27 ± 0.19^Bbc^
	0.5%	5.43 ± 0.02^Ba^	5.48 ± 0.02^Ab^	5.39 ± 0.01^BCb^	5.38 ± 0.04^Cb^	5.14 ± 0.03^Dc^
	1.0%	5.32 ± 0.02^Bb^	5.42 ± 0.02^Ac^	5.34 ± 0.02^Bc^	5.38 ± 0.05^ABb^	5.39 ± 0.05^ABab^
	1.5%	5.20 ± 0.03^CDc^	5.31 ± 0.01^Be^	5.25 ± 0.02^BCd^	5.15 ± 0.08^Dd^	5.49 ± 0.06^Aa^
	2.0%	5.18 ± 0.03^Cc^	5.37 ± 0.01^Ad^	5.25 ± 0.02^Bd^	5.27 ± 0.04^Bc^	5.39 ± 0.07^Aab^
Coliform bacteria	Control	3.69 ± 0.09^b^	ND	ND	ND	ND
	0.5%	4.21 ± 0.09^a^	ND	ND	ND	ND
	1.0%	3.54 ± 0.24^bc^	ND	ND	ND	ND
	1.5%	3.39 ± 0.09^d^	ND	ND	ND	ND
	2.0%	3.39 ± 0.09^d^	ND	ND	ND	ND

Notes

ND: not detected.

^A-E^Means sharing the same uppercase letters in the same row differ significantly (*p *<* *0.05). ^a-e^Means sharing the same lowercase letters in the same column differ significantly (*p *<* *0.05).

The initial amount of lactic acid bacteria in kimchi pastes was 5.60 log CFU/g for the untreated control and 5.14, 4.40, 4.19, and 3.42 log CFU/g for pastes containing 0.5%, 1%, 1.5%, and 2% clove powder, respectively. Thus, the amount of lactic acid bacteria was inversely related to clove powder concentration. After 10 and 20 days of storage, the population of lactic acid bacteria in the paste was 6.79 and 6.73 log CFU/g, respectively, for the control and 3.46–5.50 and 1.39–3.33 log CFU/g, respectively, for samples with 0.5%–2% clove powder. A key event in the fermentation of many foods is the conversion of sugars to lactic acid by lactic acid bacteria (Deegan, Cottera, Hilla, & Ross, 2006); inhibiting their growth could, therefore, delay the fermentation process.

The initial population of yeast and molds in kimchi pastes was 5.45 log CFU/g for the untreated control and 5.18–5.43 log CFU/g for treatment groups with 0.5%–2% clove powder; these values did not change significantly during 20 days of storage. Excessive growth of yeast and mold during kimchi paste fermentation can impede sensory satisfaction during consumption (In & Chae, 2004; Kim, Hwang, Choi, & Kim, 1992); however, there was no visible yeast and mold growth in any of our samples during fermentation.

The initial population of coliforms in kimchi pastes was 3.59 log CFU/g for the untreated control and 3.39–4.21 log CFU/g for samples treated with 0.5%–2% clove powder. In agreement with these results, clove oil has been shown to inhibit the growth of coliform bacteria in buffalo meat (Naveena et al., 2006), and a previous study found that the extracts of clove and other herbs had growth‐suppressive properties against *Listeria monocytogenes*,* Staphylococcus aureus*, and *Salmonella enterica* in raw pork (Shan, Cai, Brooks, & Corke, 2009). After 5 days of storage, there were no coliforms detected in the control or treatment groups. It has been demonstrated that the growth of coliform bacteria is inhibited by lactic acid produced by lactic acid bacteria such as *Streptococcus, Leuconostoc, and Lactobacillus* (Reinheimer, Demkow, & Candioti, 1990).

### 3.4 Changes in gas composition in packaged headspace during storage

**Figure 3:**
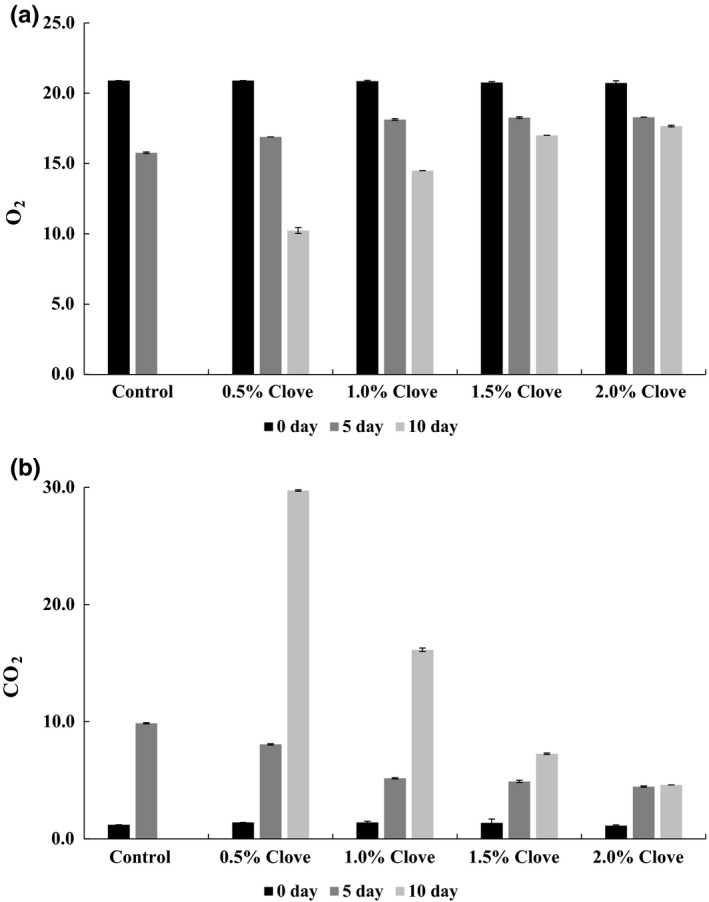
Changes in O_2_ (a) and CO_2_ (b) contents of kimchi paste treated with various concentrations of clove powder on day 0, 5, and 10 of storage at 10°C

We analyzed the gas composition in stored kimchi pastes treated with clove powder (Figure 3). The initial O_2_ concentration in the pastes was 20.73%–20.90%, with no differences observed among samples. From day 5, the O_2_ content of the container headspace increased with clove powder concentration. On day 10, the O_2_ contents for samples treated with 0.5% and 2% clove powder were 10.23% and 17.67%, respectively.

The initial CO_2_ composition in the kimchi paste container was 1.20% for the control and 1.13%–1.14% for samples treated with clove powder, with no differences among samples. From day 5, the CO_2_ content of the container headspace decreased as a function of clove powder concentration. On day 10, the CO_2_ content was 29.73% and 4.60% for samples containing 0.5% and 2% clove powder, respectively. No measurements were made of the control sample on day 10 or of any sample on days 15 and 20 because the packaging exploded as a result of gas accumulation during kimchi paste fermentation. Some lactic acid bacteria grow under aerobic conditions, producing metabolic products such as lactic and acetic acid and CO_2_ in the initial stage of fermentation, thereby increasing acidity and decreasing pH and causing CO_2_ accumulation in the kimchi packaging headspace (Meng, Lee, Kang, & Ko, 2015). Our results suggest that the presence of clove powder suppresses changes in O_2_ and CO_2_ concentration inside the kimchi paste packaging by inhibiting the growth of lactic acid bacteria.

### 3.5 Sugar content

The sugar content of kimchi pastes with various concentrations of clove powder changed during 20 days of storage at 10°C. The different uppercase letters in the row mean statistically significant (*p *<* *0.05) differences according to concentration of clove powder. The different lowercase letters in the column mean statistically significant (*p *<* *0.05) differences according to fermentation days (Table 3). Sucrose content decreased in all samples from 25,695.13–27,087.65 mg/kg on day 0 to 17,692.83–20,626.91 mg/kg on day 20. Sucrose is hydrolyzed to d‐glucose and d‐fructose by dextransucrase secreted by microorganisms such as *Lactobacillus* and *Leuconostoc* spp.; d‐fructose is utilized as an energy and carbon source, whereas d‐glucose is used for biosynthesis of dextran via polymerization (Hahn, Woo, Park, & Lee, 1997; Kim, Min, Kim, & Han, 2001).

**Table 3:** Changes in sugar content (mg/kg) of kimchi paste containing various concentrations of clove powder during fermentation at 10°C

Sample	Day	Control	0.5% Clove powder	1.0% Clove powder	1.5% Clove powder	2.0% Clove powder
Sucrose	0	27,087.65 ± 227.15^Aa^	26,167.98 ± 319.57^Ba^	26,155.63 ± 51.06^Ba^	25,695.13 ± 30.52^Ca^	26,016.58 ± 12.96^BCa^
	5	22,790.24 ± 183.86^Ab^	20,498.63 ± 184.19^Bc^	20,127.15 ± 274.99^BCc^	19,739.88 ± 87.69^Cc^	20,260.95 ± 501.45^BCb^
	10	21,487.62 ± 294.58^Bbc^	21,164.58 ± 303.85^BCb^	22,322.00 ± 240.83^Ab^	20,589.92 ± 518.01^Cb^	19,015.54 ± 132.07^Dc^
	15	20,899.00 ± 1,437.90^Bc^	20,761.91 ± 457.76^Bbc^	22,245.70 ± 164.62^Ab^	19,732.45 ± 514.43^Bc^	18,301.88 ± 117.00^Cd^
	20	20,626.91 ± 898.61^Ac^	19,073.17 ± 109.42^Cd^	19,543.29 ± 359.24^BCd^	20,350.69 ± 445.37^ABbc^	17,692.83 ± 558.24^Dd^
Glucose	0	24,175.40 ± 205.35^Cd^	24,623.20 ± 167.32^Bd^	25,016.52 ± 40.61^Ae^	24,524.23 ± 44.72^Be^	24,364.60 ± 147.63^BCd^
	5	26,778.49 ± 115.64^Ca^	27,832.13 ± 299.44^Ba^	28,669.49 ± 119.09^Ab^	27,796.58 ± 395.73^Bd^	27,836.74 ± 269.63^Bc^
	10	24,494.24 ± 326.73^Dd^	26,468.34 ± 250.48^Cb^	29,227.20 ± 133.88^Ba^	30,483.52 ± 35.77^Aa^	30,129.11 ± 79.14^Ab^
	15	24,928.72 ± 213.62^Dc^	25,217.58 ± 303.48^Dc^	28,012.06 ± 92.56^Cc^	29,183.13 ± 281.91^Bc^	30,982.16 ± 244.63^Aa^
	20	26,205.01 ± 102.90^Db^	26,433.38 ± 295.52^Db^	27,601.99 ± 104.91^Cd^	30,060.19 ± 380.57^Bb^	31,314.94 ± 487.05^Aa^
Fructose	0	28,068.15 ± 319.92^Bb^	28,318.44 ± 217.71^Bb^	28,916.22 ± 254.16^Ab^	28,360.02 ± 110.54^Bb^	28,396.77 ± 43.97^Bc^
	5	30,268.25 ± 85.06^ABa^	30,710.70 ± 325.55^Aa^	31,028.03 ± 142.36^Aa^	29,536.79 ± 757.75^Bb^	30,783.71 ± 715.16^Ab^
	10	14,141.74 ± 365.86^Dc^	18,491.83 ± 422.50^Cc^	25,327.97 ± 367.01^Bc^	31,219.19 ± 1,144.73^Aa^	31,322.40 ± 303.87^Ab^
	15	1,209.79 ± 142.90^Ed^	14,110.13 ± 349.89^Dd^	18,853.56 ± 115.83^Cd^	24,790.88 ± 412.54^Bc^	32,818.64 ± 320.15^Aa^
	20	11,957.00 ± 312.85^Ed^	13,607.37 ± 100.77^Dd^	15,421.34 ± 321.23^Cd^	21,592.28 ± 299.45^Bd^	29,279.43 ± 815.73^Ac^
Mannitol	0	1,230.45 ± 13.09^Ae^	1,247.39 ± 80.73^Ad^	1,307.78 ± 101.14^Ad^	1,294.85 ± 34.56^Ad^	1,245.06 ± 34.32^Ad^
	5	2,570.09 ± 37.30^Ad^	2,067.06 ± 252.89^Bc^	1,408.03 ± 70.42^Cd^	1,524.81 ± 129.80^Cd^	1,562.39 ± 156.00^Cc^
	10	15,878.14 ± 460.84^Ac^	12,760.55 ± 249.07^Bb^	7,555.99 ± 95.75^Cc^	3,485.68 ± 365.68^Dc^	1,720.70 ± 174.56^Ec^
	15	18,640.33 ± 108.88^Ab^	16,836.38 ± 481.81^Ba^	12,500.11 ± 216.94^Cb^	7,966.77 ± 381.03^Db^	2,484.64 ± 211.17^Eb^
	20	19,965.22 ± 297.00^Aa^	17,304.72 ± 214.75^Ba^	15,621.91 ± 35.26^Ca^	10,964.76 ± 241.57^Da^	4,304.77 ± 215.65^Ea^

Notes

ND: not detected.

^A-E^Means sharing the same uppercase letters in the same row differ significantly (*p *<* *0.05). ^a-e^Means sharing the same lowercase letters in the same column differ significantly (*p *<* *0.05).

The glucose content of kimchi pastes ranged from 24,175.40 to 25,016.52 mg/kg on day 0, with no differences among samples. After 20 days of storage, glucose content was 26,025.01 mg/kg for the untreated control and 27,601.99 and 31,314.94 mg/kg for samples treated with 1% and 2% clove powder, respectively. The fructose content of kimchi paste ranged from 28,068.15 to 28,916.22 mg/kg on day 0, with no differences observed among samples. On day 5, fructose content was highest in the untreated control sample, although the value decreased thereafter. After 20 days of storage, fructose content was 11,957.00 mg/kg for the control and 15,421.34 and 29,279.43 mg/kg for samples containing 1% and 2% clove powder, respectively. Thus, the glucose and fructose contents of kimchi pastes increased as a function of clove powder concentration.

Mannitol content in kimchi pastes ranged from 1,230.45 to 1,307.78 mg/kg on day 0, with no differences among samples. The mannitol content increased as fermentation proceeded. Among lactic acid bacteria, only hetero‐fermentative species are known to convert fructose into mannitol (Weymarn, Hujanen, & Leisola, 2002). After 20 days of storage, the mannitol content was 19,965.22 mg/kg for untreated control samples and 17,304.72, 15,621.91, 10,964.76, and 4,304.77 mg/kg for kimchi paste treated with 0.5%, 1%, 1.5%, and 2% clove powder. Thus, mannitol content decreased with increasing clove powder concentration. This may be because the clove powder inhibited the proliferation of lactic acid bacteria and consequently, mannitol production.

### 3.6 Organic acid content

Changes in the concentrations of organic acids such as lactic, acetic, citric, and malic acid in kimchi pastes upon addition of clove powder were measured. The different uppercase letters in the row mean statistically significant (*p *<* *0.05) differences according to concentration of clove powder. The different lowercase letters in the column mean statistically significant (*p *<* *0.05) differences according to fermentation days (Table 4). Lactic acid content ranged from 420.67 to 440.57 mg/kg on day 0. In untreated control samples, the lactic acid content was 440.57 mg/kg on day 0 and 9,313.93 mg/kg on day 20, indicating that lactic acid content increased as fermentation proceeded. After 10 days of storage, lactic acid content was 7,466.25 mg/kg for the control and 5,582.25, 3,394.06, 1,722.74, and 528.83 mg/kg for samples treated with 0.5%, 1%, 1.5%, and 2% clove powder. Thus, the lactic acid content decreased as clove powder concentration increased, likely due to inhibition of lactic acid bacterial growth.

**Table 4:** Changes in the organic acid content (mg/kg) of kimchi paste containing various concentrations of clove powder during fermentation at 10°C

Sample	Day	Control	0.5% Clove powder	1.0% Clove powder	1.5% Clove powder	2.0% Clove powder
Lactic acid	0	440.57 ± 9.78^Ae^	435.25 ± 11.38^ABe^	436.28 ± 6.62^ABe^	434.91 ± 6.74^ABd^	420.67 ± 10.50^Bd^
	5	2,584.48 ± 15.06^Ad^	1,711.28 ± 8.51^Bd^	731.93 ± 8.87^Cd^	472.52 ± 6.52^Dd^	471.43 ± 5.82^Dcd^
	10	7,466.25 ± 47.32^Ac^	5,582.25 ± 32.39^Bc^	3,394.06 ± 40.39^Cc^	1,722.74 ± 44.62^Dc^	528.83 ± 36.76^Ec^
	15	8,920.24 ± 27.90^Ab^	8,812.36 ± 28.33^Ab^	5,520.45 ± 37.21^Bb^	3,457.52 ± 520.93^Cb^	988.86 ± 22.35^Db^
	20	9,313.93 ± 48.23^Aa^	8,898.97 ± 34.58^Ba^	7,120.61 ± 29.94^Ca^	4,985.31 ± 17.96^Da^	2,473.76 ± 58.12^Ea^
Acetic acid	0	174.36 ± 7.63^Ee^	212.29 ± 12.54^De^	261.11 ± 2.38^Ce^	299.32 ± 7.14^Bd^	346.19 ± 16.19^Ad^
	5	1,447.14 ± 65.10^Ad^	819.93 ± 1.95^Bd^	360.30 ± 18.82^Cd^	350.63 ± 10.33^Cd^	407.57 ± 4.58^Ccd^
	10	4,892.61 ± 34.20^Ac^	3,303.11 ± 21.89^Bc^	1,730.04 ± 20.61^Cc^	886.23 ± 62.17^Dc^	470.15 ± 75.62^Ec^
	15	5,815.19 ± 29.16^Ab^	5,272.96 ± 19.01^Ba^	2,923.75 ± 31.20^Cb^	1,903.02 ± 32.37^Db^	553.02 ± 10.54^Eb^
	20	6,094.36 ± 33.68^Aa^	5,135.41 ± 59.69^Bb^	3,626.73 ± 21.89^Ca^	2,499.72 ± 6.90^Da^	1,295.10 ± 36.76^Ea^
Citric acid	0	5,772.29 ± 1.33^Aa^	5,749.76 ± 1.83^Ba^	5,563.09 ± 2.18^Cb^	5,550.50 ± 9.97^Da^	5,418.83 ± 7.96^Eb^
	5	3,875.21 ± 15.27^Eb^	5,060.92 ± 1.81^Db^	5,589.88 ± 5.49^Aa^	5,552.16 ± 3.49^Ba^	5,452.25 ± 1.04^Cab^
	10	1,492.34 ± 18.11^Ec^	3,581.71 ± 46.18^Dc^	5,318.61 ± 24.84^Cc^	5,632.34 ± 66.21^Aa^	5,527.17 ± 6.51^Ba^
	15	566.88 ± 1.14^Ee^	1,017.35 ± 42.40^De^	5,149.59 ± 4.83^Cd^	5,325.63 ± 81.81^Bb^	5,406.95 ± 1.82^Ab^
	20	737.09 ± 3.58^Ed^	1,740.74 ± 2.17^Dd^	4,671.88 ± 2.31^Ce^	5,162.52 ± 4.60^Bc^	5,546.86 ± 115.00^Aa^
Malic acid	0	3,854.13 ± 111.73^A^	3,663.03 ± 37.52^Ba^	3,404.34 ± 82.98^Ca^	3,319.29 ± 32.65^Ca^	3,376.38 ± 107.35^Ca^
	5	2,376.93 ± 90.45^A^	2,356.49 ± 22.86^Ab^	3,168.23 ± 74.74^Bb^	3,354.04 ± 64.75^Ca^	3,434.33 ± 57.29^Ca^
	10	ND	1,242.63 ± 29.78^Dc^	1,678.30 ± 24.34^Cc^	2,268.49 ± 22.55^Bb^	2,968.85 ± 143.70^Ab^
	15	ND	ND	ND	ND	2,500.54 ± 65.22^c^
	20	ND	ND	ND	ND	1,887.22 ± 65.60^d^

Notes

ND: not detected.

^A-E^Means sharing the same uppercase letters in the same row differ significantly (*p *<* *0.05). ^a-e^Means sharing the same lowercase letters in the same column differ significantly (*p *<* *0.05).

The initial acetic acid content of kimchi paste was 174.36 mg/kg for untreated control samples and 212.29, 261.11, 299.32, and 346.19 mg/kg for those treated with 0.5%, 1%, 1.5%, and 2% clove powder, respectively, indicating that acetic acid content increased with addition of clove powder. In untreated control samples, the acetic acid content increased from 174.36 mg/kg on day 0 to 6,094.36 mg/kg on day 20. After 10 days of storage, the value was 4,892.61 mg/kg for the control group and 3,303.11, 1,730.04, 886.23, and 470.15 mg/kg for samples treated with 0.5%, 1%, 1.5%, and 2% clove powder, respectively.

The citric acid content of kimchi pastes ranged from 5,418.83 to 5,772.29 mg/kg on day 0; the value in the untreated control increased from 5,772.29 mg/kg on day 0 to 737.09 mg/kg on day 20, indicating that citric acid content decreased as fermentation proceeded. After 20 days of storage, citric acid content was 737.09 mg/kg for the control as compared to 1,740.74, 4,671.88, 5,162.52, and 5,546.86 mg/kg for samples treated with 0.5%, 1%, 1.5%, and 2% clove powder, respectively. Lactic acid bacteria produce acetic acid during citric acid metabolism (Panagou, Schillingerb, Franzb, & Nychasa, 2008). Our results indicate that the citric acid content of kimchi pastes increased with the addition of clove powder, which likely slowed the metabolism of citric acid to acetic acid by suppressing lactic acid bacterial growth.

The malic acid content of kimchi pastes was 3,319.29–3,854.13 mg/kg on day 0. The value in untreated control samples decreased sharply as fermentation proceeded; after 10 days of storage, there was no malic acid detected. This may be because lactic acid bacteria accelerated the transformation of malic acid to lactic acid and acetic acid as they proliferated during kimchi paste fermentation (Lim et al., 2013; Panagou et al., 2008). In samples treated with 0.5%, 1%, 1.5%, and 2% clove powder, the malic acid contents was 1,242.63, 1,678.30, 2,268.49, and 2,968.85 mg/kg, respectively, after 10 days of storage; however, there was no malic acid detected after 15 days of storage in the 0.5%, 1%, and 1.5% clove powder treatment samples. On the other hand, malic acid content was 3,376.38, 2,968.85, and 1,887.22 mg/kg on day 0, 10, and 20 of storage for samples containing 2% clove power.

### 3.7 Changes in pH, titratable acidity, and reducing sugar content during storage

**Figure 4:**
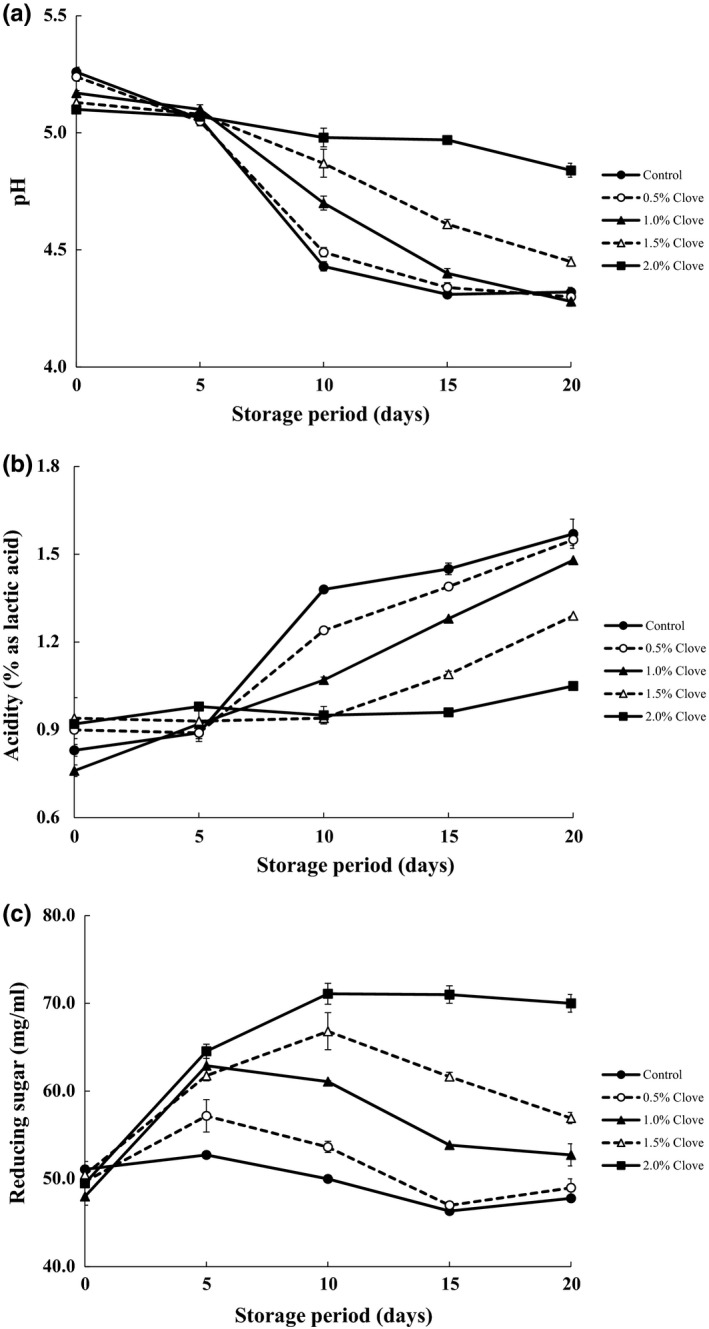
Changes in pH(a), titratable acidity(b), and reducing sugar content(c) of kimchi paste containing various concentrations of clove powder during 20 days of storage at 10°C

Changes in pH, titratable acidity, and reducing sugar content of kimchi pastes containing various concentrations of clove powder during 20 days of storage at 10°C were analyzed (Figure 4). The pH and acidity are important factors determining the maturity and quality of kimchi and kimchi pastes; an important step in their fermentation is the transformation of sugar to lactic and other organic acids by the lactic acid bacteria, which reduces the pH and increases acidity (Jeong et al., 2011; Kang et al., 2015). Before storage and immediately after addition of clove powder, the pH of kimchi pastes was 5.10–5.29, with no differences among samples. The pH of the untreated control sample decreased from 5.29 on day 0 to 4.32 on day 20, whereas samples containing 2% clove powder showed a decrease in pH from 5.10 to 4.84 at these time points. This indicated that adding clove powder delayed the decrease in pH. Accordingly, acidity increased from 1.38% on day 0 to 1.57% on day 20 in control samples and from 0.92% to 1.05% in samples containing 2% clove powder. Clove extracts have been used as natural preservative in raw pork (Shan et al., 2009) since they contain high levels of phenolic compounds that not only extend shelf life, but also maintain meat color via antioxidant activities.

The reducing sugar content of kimchi paste increased until day 10 before decreasing, demonstrating that additional clove powder produces smaller changes; these are related to the decrease in pH and increase in acidity, and probably result from the decomposition and utilization of reducing sugars as a carbon source for microbial growth during fermentation (Afoakwa, Kongor, Takrama, & Budu, 2013; Kang et al., 2015).

## 4 CONCLUSIONS

This study investigated the possibility of using clove powder as a natural additive in fermentation to improve the longevity of kimchi paste. The presence of clove powder significantly reduced the population of total aerobic and lactic acid bacteria in fermented kimchi paste, delaying changes in O_2_ and CO_2_ concentration, pH, titratable acidity, and reducing sugar content during storage. These results suggest that adding clove powder to kimchi paste can extend its shelf life without compromising other attributes.

## CONFLICT OF INTEREST

The authors declare that they do not have any conflict of interest.

## ETHICAL REVIEW

This study does not involve any human or animal testing.
